# Reiki for decrease postmenopausal symptoms and depression: a randomized clinical trial

**DOI:** 10.1590/1806-9282.20250076

**Published:** 2025-08-08

**Authors:** Esra Sabancı Baransel, Tuba Uçar

**Affiliations:** 1 Inonu University, Faculty of Health Sciences, Department of Midwifery – Malatya, Turkey.

**Keywords:** Reiki, Menopause, Signs and symptoms, Depression

## Abstract

**OBJECTIVE:**

The aim of this study was to determine the effects of Reiki applied to women in the postmenopausal period on menopausal symptoms and depression levels.

**METHODS:**

This randomized controlled study was conducted with postmenopausal women registered in a family health center. The sample of the study consisted of 82 women (Reiki=41, control=41). While four sessions of Reiki were applied to the women in the Reiki group, once a week for 4 weeks. All participants in the control group received routine care provided by health professionals at the family health center. The Menopause Rating Scale and Beck Depression Inventory were used to collect data. The data were analyzed using SPSS 25.0, with independent and dependent t-tests, and effect sizes were calculated using Cohen's d. The analysis was conducted using the per-protocol approach, where only participants who fully completed the intervention and adhered to the protocol were included in the analysis.

**RESULTS:**

The mean scores of menopausal complaints (17.31 vs. 21.73; p<0.01), somato-vegetative complaints (2.70 vs. 3.85; p<0.01), and psychological complaints (10.07 vs. 12.60; p<0.05) were significantly reduced in the Reiki group compared to the control group. Similarly, the mean score of depression (9.63 vs. 15.90; p<0.001) was significantly decreased in the Reiki group compared to the control group.

**CONCLUSION:**

Reiki practice significantly reduced menopausal symptoms and depression levels in postmenopausal women. These findings suggest that Reiki may be an effective complementary treatment option for women going through menopause.

## INTRODUCTION

With the increase in the life expectancy of women, the time spent after menopause has increased. Therefore, women spend more than one-third of their lives in the menopause period^1,2^. Menopause is due to the hormonal changes that occur; menopause is considered a special period in which many physical and mental changes are experienced^3,4^. During this period, women may have somatic complaints such as hot flashes, night sweats, palpitations, joint and muscle disorders, psychological complaints, fatigue, irritability, depression, forgetfulness, difficulty in concentration, and urogenital complaints, such as atrophy in the genitals, dryness, dyspareunia, decreased sexual desire, and urinary system infections^5,6^. These complaints may significantly reduce women's mental, physical, and social quality of life, and negatively affect their social relationships, daily activities, and productivity mildly or severely in the professional sense^7-9^. These negativities also bring along psychological disorders such as uncontrollable stress, anxiety, and depression.

Studies in the literature show that Reiki leads to a decrease in symptoms related to hypertensive diseases, anxiety, depression, sleep disorders, emotional disorders, and the menstrual^9-15^. A systematic review examining the efficacy of Reiki reported that no firm conclusions could be drawn about the effectiveness of Reiki with the available evidence and that more randomized controlled trials are needed^16^. Despite the reported positive effects, the exact mechanisms for these effects are still unknown. However, the number of studies addressing the effects of Reiki during menopause is still insufficient to integrate the findings into the body of knowledge to advance evidence-based practice. Therefore, in this study, it was aimed to determine the effects of Reiki administered to postmenopausal women on menopausal symptoms and depression levels.

## METHODS

### Design

This study was designed as a randomized controlled trial in which participants were randomly assigned to Reiki or control groups. The study was conducted with postmenopausal women registered at a Family Health Center (FHC) located in the center of a province in eastern Türkiye between May 2023 and July 2024. Women who applied to the FHC for any reason, agreed to participate voluntarily, and met the inclusion criteria were included in the study. The criteria for inclusion in the study were as follows: those who were between the ages of 45 and 55, did not have hearing problems, did not have any psychiatric diseases, were in a natural menopause period, and did not take hormone replacement therapy.

A web-based software was used to determine the sample size. A priori power analysis was performed to estimate the appropriate sample size. In the literature, the mean score of menopausal symptoms, which is the primary result of the study, was found to be 16.6 (standard deviation 8.9)^17^. The sample size was calculated as 41 for each group, assuming a 5% error level, bidirectional significance level, 95% confidence interval, 80% representation of the population, and assuming to create a decrease in the post-intervention menopausal symptoms mean score by 5 points. Permission was obtained from the Inonu University Non-Interventional Clinical Research Ethics Committee (Decision number: 2023/4577) to conduct the study.

### Data collection tools

In the study, data were collected with a sociodemographic questionnaire including questions about age, last menstrual period, education level, Menopause Rating Scale (MRS), Beck Depression Inventory (BDI), and a question investigating the level of being affected by menopause complaints. The question investigating the level of being affected by menopause is: "At which level do menopause complaints affect you?". Women answered this question on a scale of 0 (very little/very slightly) to 10 (extreme/very much).

MRS was developed to assess the severity of menopause symptoms. The scale consists of 11 items and 3 subscales. These three subscales are called somato-vegetative complaints (such as night sweating and hot flush), psychological complaints (such as anxiety and depression), and urogenital complaints (such as vaginal dryness). An increase in the total score obtained from the scale indicates that the menopausal complaints experienced are intensified. The Cronbach's alpha reliability coefficient of the scale was calculated as 0.84^18^. In this study, the Cronbach's alpha reliability coefficient of the scale was determined as 0.80.

BDI is used to have information about depressive symptoms and attitudes by evaluating the findings of emotional, cognitive, and motivational symptoms that can be seen in depression. A high total score indicates a high level of depression severity. The Cronbach's alpha reliability coefficient of the scale was determined as 0.80^19^. In this study, the Cronbach's alpha reliability coefficient of the scale was determined as 0.88.

### Data collection

In the study, 4 weeks after the data collection tools used in the research were filled in as a pre-test, post-test data were obtained with the same measurement tools.

In the Reiki group, Reiki was applied by a researcher certified in First Degree Reiki (Reiki I). The intervention followed the standard protocol for Reiki I, which involves "aura attunement" through either light touch or holding the hands a few centimeters above the body. The practitioner sent energy to each chakra or specific body area for 3–5 min. Before starting each session, the practitioner and participant removed any jewelry to minimize distractions. The participant was positioned comfortably, either sitting or lying down, with arms and legs open to avoid physical contact.

Each Reiki session lasted 30–40 min and was conducted individually once a week for 4 weeks. The practitioner ensured a calm environment by dimming the lights and avoiding external distractions, creating a serene atmosphere conducive to healing. After each session, the practitioner washed their hands to maintain hygiene, as per Reiki protocol. Participants in the control group did not receive any intervention, and only routine care services such as general health screenings and psychological counseling provided by the FHC were provided. In addition, women were offered medical support for menopause-related complaints, such as hormone therapy and nutritional advice, by the services typically available at the FHC.

### Statistical analysis

The data obtained in the study were analyzed using the SPSS 25.0 program. In the study, numerical data were shown as mean and standard deviation, and nominal data (demographic) as frequency and percentage. The chi-square test was used for the comparison of nominal data between groups. In the evaluation of numerical data, first, the Kolmogorov-Smirnov test was used to determine whether the variables met the condition of showing normal distribution. Since the data showed a normal distribution, an independent samples t-test was used for the comparison of the two groups, and a paired samples t-test was used for intergroup comparisons. If the results of the t-tests were significant, effect sizes were computed using Cohen's d to identify significant differences. The results were evaluated at a p<0.05 significance level. For the analysis, the per-protocol approach was applied, and only those participants who fully completed the intervention and adhered to the protocol were included in the analysis.

## RESULTS

**Figure 1:**
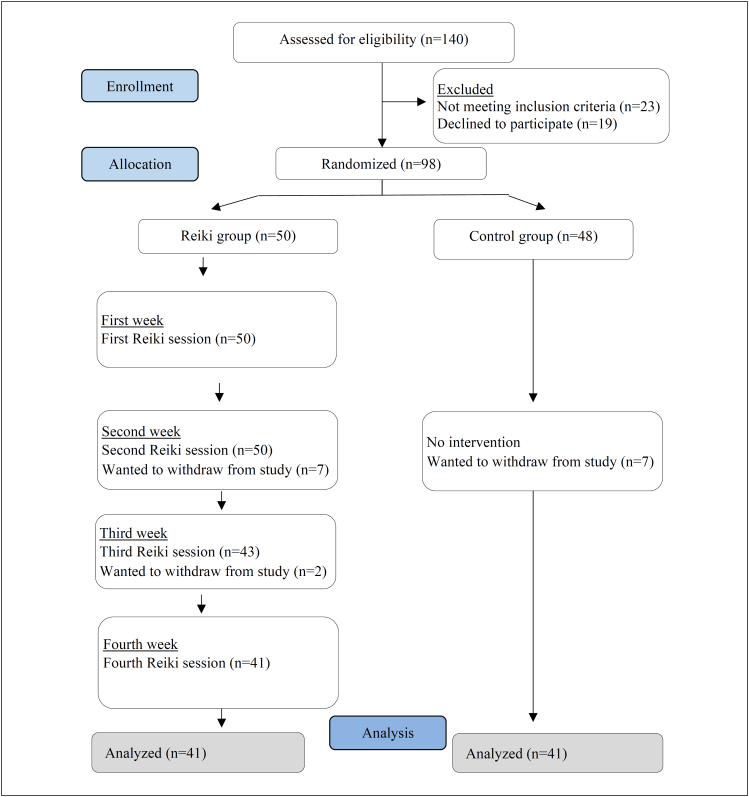
Consolidated Standards of Reporting Trials (CONSORT) diagram of the participants for each stage in this study.

The research was completed with a total of 82 participants. Each stage of the registration of the participants is given in detail in Figure 1. It was determined that the sociodemographic characteristics (age, the age when the last menstrual period was experienced, educational level, and income) of the participants in the Reiki and control groups were similar (p>0.05).

Table 1 shows the pre-test and post-test mean scores of the BDI, MRS, and subscales of the participants. There was no difference between the pre-test mean scores of the Reiki and control groups (p>0.05). Post-test mean scores of menopausal complaints were significantly lower in the Reiki group than in the control group (17.31 vs. 21.73; p<0.01); the effect size was large (η^2^=0.747). Post-test mean scores for the MRS's subscales (somato-vegetative complaints and psychological complaints) were also significantly lower in the Reiki group compared to the control group, and the effect sizes were large (η^2^=0.856, η^2^=0.665, respectively). When the post-test depression mean scores were compared, it was significantly lower in the Reiki group (9.63 vs. 15.90; p<0.001); the effect size was large (η^2^=1.023).

**Table 1:** Comparison of the mean scores of the Beck Depression Inventory, Menopause Rating Scale, and its subscales of the Reiki and control groups.

		Reiki group (n=41)		Control group (n=41)		Test^a^ and p-value		Effect size (d)
		Mean±SD		Mean±SD				
Mean age/year		49.36±3.03		49.97±3.16				
**Somato-vegetative complaints subscale**								
	Pre-test	4.26±1.43		3.56±1.96		t=1.864	p=0.066	
	Post-test	2.70±0.92		3.85±1.66		t=-3.847	**p<0.001**	0.856
**Test** b **and p-value**		t=7.247	**p<0.001**	t=-0.710	p=0.482			
**Psychological complaints subscale**								
	Pre-test	12.14±3.59		12.39±5.66		t=-0.233	p=0.816	
	Post-test	10.07±2.66		12.60±4.67		t=2.101	**p=0.003**	0.665
**Test** b **and p-value**		t=3.715	**p=0.001**	t=-0.192	p=0.849			
**Urogenital complaints subscale**								
	Pre-test	4.51±2.20		4.82±2.72		t=-0.579	p=0.564	
	Post-test	4.53±1.45		5.26±2.94		t=-1.429	p=0.157	
**Test** b **and p-value**		t=-0.067	p=0.947	t=-0.750	p=0.458			
**MRS total**								
	Pre-test	20.92±5.42		20.78±9.10		t=-0.088	p=0.930	
	Post-test	17.31±3.97		21.73±7.36		t=-3.377	**p=0.001**	0.747
**Test** b **and p-value**		t=4.733	**p<0.001**	t=-0.522	p=0.604			
**BDI**								
	Pre-test	14.48±7.04		14.58±9.37		t=-0.053	p=0.958	
	Post-test	9.63±3.67		15.90±7.85		t=-4.629	**p<0.001**	1.023
**Test** b **and p-value**		t=5.167	**p<0.001**	t=-0.745	p=0.461			

MRS: Menopause Rating Scale, BDI: Beck Depression Inventory, SD: standard deviation,

a Independent samples t-test;

b Paired samples t-test;

Cohen's d effect sizes were reported only when the differences were significant. Bold values indicate statistically significant differences (p < 0.05) and their corresponding effect sizes (Cohen's d) where applicable.

**Figure 2:**
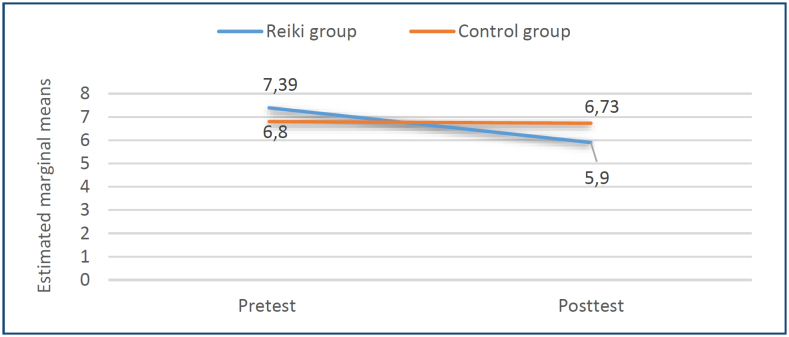
The pre–post-test score curves for Reiki and control groups to be affected by menopause complaints.

Figure 2 shows the pre-test and post-test score curves for Reiki and control groups to be affected by menopause complaints. There was no difference between the scores of the pre-test menopause complaints of the Reiki and control groups (t=1.453, p=0.150). The difference between the groups in terms of the post-test scores of being affected by menopausal complaints was found to be statistically significant in favor of the Reiki group (t=-2.192, p=0.031).

## DISCUSSION

The results of this study presented that Reiki administered to postmenopausal women may be a promising approach to alleviate menopausal symptoms and depression symptoms, including somatic and psychological complaints.

Menopausal symptoms were significantly reduced in the Reiki group participants compared to those in the control group. In addition, somatic and psychological complaints decreased significantly after the application of Reiki compared to the control group. During Reiki practice, a focused effect occurs on the body through certain electromagnetic waves. Thus, it starts the physical and mental healing process by solving the negative energy blockages in the body^14,15,20,21^. These results in the study support the idea that Reiki practice may be the result of balancing the organism and supporting the full functioning of the organism. Although there is limited scientific research on the effects of Reiki, it was suggested to promote relaxation and improve overall well-being^22^. In a study conducted to evaluate the effects of Reiki applications on menopausal symptoms, it was found that it reduced somatic, psychological, and urogenital symptoms in women, and this effect was long-lasting^23^. These findings showed that Reiki makes a positive contribution to the menopausal period by helping women cope with the symptoms in the postmenopausal period. Another important result of the current study is the positive effect of Reiki on alleviating depression in postmenopausal women. After the intervention, the depression levels of the women in the Reiki group decreased significantly compared to the women in the control group.

Reiki was associated with the ability to increase psychological well-being and improve overall quality of life^22^. Studies have generally suggested that Reiki alleviates depression^22-25^. A previous study revealed that relaxation was achieved and depression scores were significantly reduced during Reiki applied to elderly individuals^25^. Additionally, another study included 1,411 participants to measure the effect of a single session of Reiki on physical and psychological health^20^. This study showed that Reiki improves physical and psychological symptoms associated with many health conditions, including mood, depression, anxiety, fatigue, lethargy, nausea, shortness of breath, appetite, and general well-being^20^. This study has some limitations. First, the results of this study cannot be generalized because it was conducted only in one center. Second, the lack of determination of the long-term effects of Reiki practice on menopausal symptoms and depression is another limitation. Third, the study was conducted with a limited number of participants and without the Sham Group, which might have led to bias. The results of this study can be considered as a pilot study to determine future therapeutic assumptions. Despite these limitations, it is thought that this study would lead to future studies to reduce the level of depression and menopausal symptoms of Reiki applied to women in the postmenopausal period.

## CONCLUSION

According to the results of the research, Reiki applied to women in the postmenopausal period provided positive outcomes regarding menopausal symptoms and depression levels, including somatic and psychological complaints. In this direction, it is recommended to use Reiki as an alternative treatment method in addition to pharmacological methods in the treatment of common psychological and somatic complaints during menopause.

## Data Availability

The datasets generated and/or analyzed during the current study are available from the corresponding author upon reasonable request.
